# Histological Microstructure and In Vitro Mechanical Properties of Postmenopausal Ewe Perineal Body With and Without Estrogen Replacement Therapy

**DOI:** 10.1007/s00192-026-06692-7

**Published:** 2026-06-08

**Authors:** Petra Kochova, Lucie Hajkova Hympanova, Katerina Mackova, Robert Cimrman, Zbynek Tonar, Jan Deprest, Vladimir Kalis

**Affiliations:** 1 European Centre of Excellence NTIS, Faculty of Applied Sciences, University of West Bohemia, Pilsen, Czech Republic; 2 Centre for Surgical Technologies, KU Leuven, Leuven, Belgium; 3 Department of Development and Regeneration, KU Leuven, Leuven, Belgium; 4 The Institute for the Care of Mother and Child, Third Faculty of Medicine, Charles University, Prague, Czech Republic; 5 Department of Gynecology, Obstetrics and Neonatology, General University Hospital in Prague and First Faculty of Medicine, Charles University, Prague, Czech Republic; 6 New Technologies Research Centre NTC, University of West Bohemia, Pilsen, Czech Republic; 7 Department of Histology and Embryology and Biomedical Center, Faculty of Medicine in Pilsen, Charles University, Prague, Czech Republic; 8 Pelvic Floor Unit, University Hospitals KU Leuven, Leuven, Belgium; 9 Department of Gynecology and Obstetrics, University Hospital, Pilsen, Czech Republic; 10 Faculty of Medicine in Pilsen, Charles University, Prague, Czech Republic

**Keywords:** Biomechanics, Menopause, Pelvic floor, Perineal body, Sheep model, Stereology

## Abstract

**Introduction and Hypothesis:**

The objective was to assess the regional variability, mechanical properties, and histological composition of the perineal body in postmenopausal ewes with and without delayed estrogen replacement therapy (ERT), providing data relevant for pelvic floor modeling and the understanding of menopausal tissue changes.

**Methods:**

The fibromuscular layer of the central tendon of the perineal area between the rectum and vagina was used for the study. We analyzed 24 specimens from 8 postmenopausal ewes with ERT (initiated 180 days after ovariectomy and continued for 100 days)—POST-ERT group—and 21 specimens from 7 ewes without ERT—POST group, all 280 days after ovariectomy. Uniaxial tensile tests determined the Young’s moduli in small (E_0_) and large (E_1_) deformation regions, ultimate stress, and ultimate strain. Histological stereology quantified volume fractions of collagen, elastin, smooth muscle, skeletal muscle, and adipose tissue in three regions (R1–R3). Group comparisons and regional analyses were performed using nonparametric statistics.

**Results:**

Mechanical parameters did not differ significantly between groups (E_0_: 28/25 kPa; E_1_: 0.38/0.33 MPa; ultimate stress: 0.23/0.23 MPa; ultimate strain: 0.94/0.90 for POST-ERT/POST). ERT was associated with higher collagen (33% versus 23%, *p* < 0.001) and elastin (6% versus 5%, *p* < 0.01) content. Regional differences were minimal, with only modest variation in adipose and smooth-muscle fractions and ultimate stress between the midline and lateral regions.

**Conclusions:**

Despite prolonged estrogen deprivation, the perineal body maintained mechanical integrity. Structural changes, particularly increased collagen and elastin after delayed ERT, suggest adaptive remodeling but do not translate into measurable biomechanical reinforcement within the studied timeframe. The results provide parameters for finite element modeling of pelvic floor disorders.

**Supplementary Information:**

The online version contains supplementary material available at https://doi.org/10.1007/s00192-026-06692-7.

## Introduction

The perineum, a highly complex fibromuscular structure, is located between the posterior fourchette and the anal sphincter. The perineal body is a crucial component of the perineum that forms the central attachment of the perineal muscles and supports the lower part of the pelvic floor [1]. During delivery, the perineal body plays a key role, but is also susceptible to damage, such as tearing and stretching. Damage to this central attachment can lead to prolapse of the pelvic viscera [2]. Despite its significance, the perineal body is not commonly included in finite element models of the pelvic floor [3–6]. A major limitation is the lack of the real mechanical parameters of the perineal body. Although studies addressing the mechanics of the perineum are limited, an increasing number of studies have been published over the past decade [7–9]. This trend is expected to enhance the inclusion of the perineal body in mathematical models, thereby facilitating more precise predictions regarding prolapse of the pelvic floor, mechanical function of pelvic floor support, and mesh insertion to support the pelvic floor.

Based on the study focusing on the perineal body of postmenopausal ewes 150 days after ovariectomy [10], we extend the evaluation of the postmenopausal mechanical and structural changes in the perineal body to 280 days after ovariectomy. We hypothesized that stiffness and ultimate stress would decrease over time following ovariectomy, and that this decrease would be associated with structural changes caused by hormonal deprivation. We also supposed that delayed estrogen replacement therapy (ERT) could reverse these potential changes. Therefore, this study also assesses the effect of delayed ERT, initiated 180 days after ovariectomy to simulate treatment initiated later after the onset of human menopause. Two groups of postmenopausal ewes, without or with ERT for the last 100 days, were used for this purpose. ERT is used to regulate the decline of estrogen level after menopause and has been reported to improve vaginal health and pelvic floor muscle strength [11, 12].

Adult ewes are often preferred to other large quadrupeds for pelvic floor studies [13–16]. The ewe pelvis, vaginal dimensions, and pelvic connective tissue organization are more comparable with those of women, facilitating biomechanical testing, perineal and vaginal repair, and mesh implantation using clinically relevant techniques. Ewes can also develop spontaneous pelvic organ prolapse (POP) associated with pregnancy and vaginal delivery, reflecting key human risk factors. We selected the ewe animal model owing to its comparable pelvic anatomical structure with humans and its spontaneous development of POP related to pregnancy and vaginal birth [17].

The primary objectives of the study were as follows:

1. To determine the regional mechanical variability of Young’s modulus of elasticity in both the small (E_0_) and large (E_1_) deformation regions, as well as the ultimate stress and ultimate strain along the perineal body.
2. To determine the regional structural variability of the volume fractions of collagen, elastin, smooth muscle, skeletal muscle, and adipose tissue along the perineal body.
3. To identify the mechanical and structural differences in perineal tissue between postmenopausal ewes with and without ERT.

## Materials and Methods

### Specimen Collection and Preparation

Eight postmenopausal ewes with ERT (POST-ERT group, 8.0 ± 0.8 years old, parity 1.63 ± 0.52, weight 60.1 ± 10.4 kg (mean ± standard deviation)) and seven postmenopausal ewes without ERT (POST group, 8.5 ± 1.8 years old, parity 1.57 ± 0.53, weight 65.7 ± 6.4 kg (mean ± standard deviation)) were used for the study. Both groups were evaluated 280 days after surgical ovariectomy.

The ERT for the POST-ERT group began 180 days after ovariectomy and continued for 100 days. An estradiol implant in the form of a sustained-release system was inserted under the skin in the inguinal region. This system was made from silastic tubing (inside diameter: 0.335 cm; outside diameter: 0.465 cm; Dow Corning, Midland, MI, USA) and was filled with pure crystalline 17β-estradiol (E2). It was sealed on both ends with silicone glue (732 multipurpose sealant; Dow Corning). The capsule was 5 cm long (with a 0.5-cm glue plug on each end). When placed under the skin, the capsule has been shown to maintain a physiological serum E2 concentration, producing a serum E2 level of 5 pg/ml at a release rate of approximately 1.5 pg/day. This level was selected to reverse menopausal changes in ewes and restore normal physiological estrogen levels [18, 19]. Prior to insertion, the implants were equilibrated in a 0.1% albumin buffer solution containing 2% fetal bovine serum in phosphate-buffered saline at 37 °C for 24 h to prevent a transient post-insertion peak in circulating steroids [20].

The ewes were euthanized by intravenously administering pentobarbital (20 ml/50 kg; Release®, Ecuphar, Oostkamp, Belgium). The entire perineal area between the rectum and vagina was excised to assess potential differences along the perineal body. Only the fibromuscular layer of the central tendon was used for the study; the subcutaneous tissue and the skin were excised. The tissue was divided into three regions of the perineal body: the midline region (R2), and the sagittal sections 5 mm to the left (R1) and 5 mm to the right (R3; anatomical orientation) as in Kochová et al. [10].

We used 24 POST-ERT group specimens and 21 POST group specimens for the mechanical testing. Additionally, we used 22 POST-ERT group and 21 POST group neighboring specimens for the histological analysis. The specimens used for the mechanical testing were measured immediately after being excised from the animal.

### Mechanical Measurements

We followed the mechanical protocol published for postmenopausal women and pregnant/postmenopausal ewes in Kochová et al. [9, 10]. The mechanical testing provided the following key parameters: Young’s modulus in the small and large deformation regions, as well as ultimate stress and strain.

Young’s modulus (tissue stiffness) reflects a tissue’s resistance to stretching. Higher values indicate stiffer, more supportive tissue (e.g., a firmer perineal body), whereas lower values indicate greater compliance. The small deformation region represents elastic, reversible stretching under everyday loads (e.g., normal increases in intra-abdominal pressure). Beyond this elastic range, during events such as childbirth, heavy lifting, or surgery, tissues enter the large deformation region, where micro-tears or remodeling may occur. Ultimate stress is the maximum load before tissue failure. Higher values indicate stronger tissue. Ultimate strain is the maximum elongation before failure, and higher values indicate greater extensibility.

The dimensions of the relaxed tissue specimens were measured immediately after their excision from the body by a single person (KM) using a digital caliper and are shown in Supplement 1. Only a subset of the entire excised perineal area between the rectum and vagina was measured; specifically, we measured the dimensions of the three pieces of the perineal body belonging to the midline and sagittal regions. The average thickness and average width of the specimens was 5.6 mm and 11.7 mm respectively. The specimens were clamped into a Zwick/Roell Z050 traction machine (Zwick/Roell, Ulm, Germany) for testing. The initial length of each specimen in the transverse direction between the clamps was preset to 10 mm. Uniaxial tensile loading was applied perpendicular to the midline of the perineal body at room temperature.

Each specimen underwent 20 preconditioning cycles with elongations up to 15% of the initial length. Then, linear deformation loading continued until the perineal body ruptured. The loading velocity was 6 mm/min. Deformation and related force were recorded during the uniaxial loading to generate stress–strain curves. Mechanical properties (Young’s modulus, ultimate stress, and ultimate strain) were then derived from these curves. Stress was calculated as the actual force divided by the original cross-sectional area (width × thickness), and strain was calculated as the actual elongation divided by the initial length. We used the least-squares method to determine the Young’s modulus from the stress–strain curve in the small deformation region (0–18%, depending on the shape of the curve) and the large deformation region (20–150%, depending on the shape of the curve). We determined all mechanical parameters using the open-source software ElfPy (https://github.com/rc/elfpy).

Ultimate stress and ultimate strain were determined at the point of perineal body rupture, which is not necessarily the point of highest stress on the stress–strain curve. This point is visible on the stress–strain curve as a rapid decrease in stress.

### Histology and Microscopic Quantification

We performed the structural analysis according to the protocol for postmenopausal women and pregnant/postmenopausal ewes published in Kochová et al. [9, 10].

**Fig. 1:**
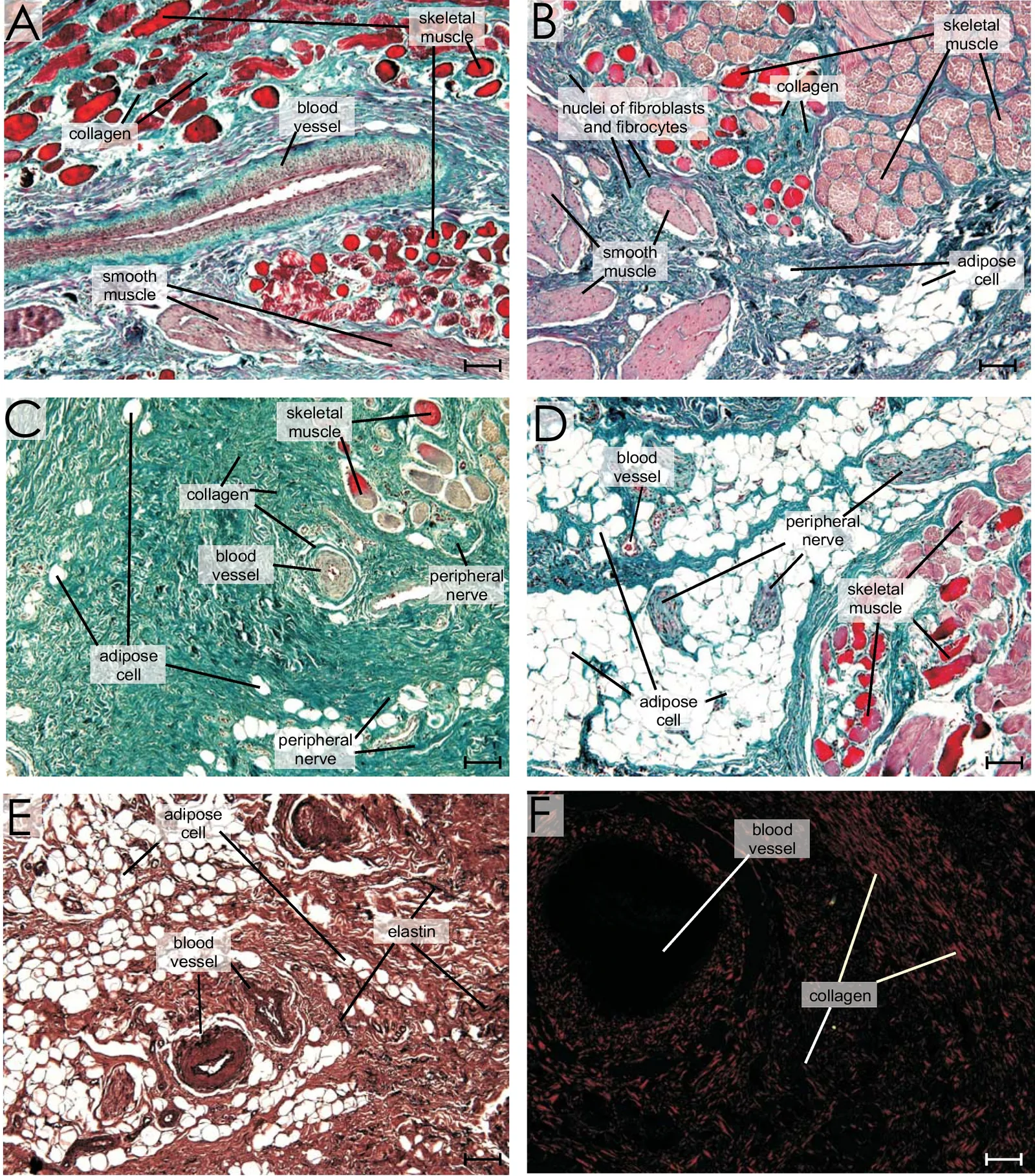
Structure of the ewe perineal body. The skeletal and smooth-muscle bundles were embedded in collagen tissue (**A**–**D**). The bundles of skeletal and smooth muscle were often close together (**A**). Elastin was present in the areas around or between adipose cells or in the dense connective tissue (**E**). Adipose cells were grouped in clusters surrounded by or crossed by collagen and elastin fibers (**B**, **D**) or individually embedded in connective tissue (**C**, **E**). The residual tissue consisted of blood vessels, peripheral nerve cells, fibroblasts, and fibrocytes. The images were stained with Verhoeff’s hematoxylin and green trichrome (**A**–**D**), orcein (**E**), and picrosirius red under polarized light (**F**). Scale bar = 100 μm

The tissue specimens reserved for structural quantification underwent standard histological processing, including formalin fixation and paraffin embedding. The specimens were then cut into vertical uniform random (VUR) serial sections (5 μm) using a Leica RM 2135 microtome (Leica Microsystems GmbH, Wetzlar, Germany) [21]. In brief, the cutting plane was oriented perpendicular to the tissue surface (defining the vertical axis) and the block was then rotated around this axis by a random angle prior to sectioning to avoid preferential in-plane orientation [22]. The body surface used to define the vertical axis was standardized based on the anatomical orientation of the excised perineal body. After excision of the perineal region between the rectum and vagina, the specimen retained identifiable anatomical landmarks, including the vaginal side, rectal side, and the midline of the perineal body. The distinction between the original external (cutaneous) surface and the deeper aspect of the excision was always unambiguous. The external perineal surface, corresponding to the plane between the posterior vaginal wall and anterior rectal wall, was used as the reference plane for orientation. Prior to embedding, specimens were aligned so that this surface was parallel to the base of the embedding cassette, ensuring consistent definition of the vertical axis across samples. The sections were then stained using three methods: Verhoeff’s hematoxylin and green trichrome stain to visualize the smooth and skeletal muscle (Fig. 1A–D); the orcein stain to visualize the elastin and adipose cells (Fig. 1E); and the picrosirius red stain to visualize the collagen (i.e., free collagen and the collagen of the adventitia of the blood vessels) in polarized light (Fig. 1F). We detected uneven staining of skeletal muscle fibers in some specimens (e.g., Fig. 1B–D). In green trichrome methods used to demonstrate muscle and collagen, the final cytoplasmic hue depends on competitive binding and displacement of acid dyes during differentiation steps (polyacid/acetic acid), and minor local variations within a section (micro-variation in thickness, reagent coverage/flow, hydration and differentiation intensity) can lead to fiber-to-fiber differences in red intensity, occasionally giving a paler or grayish appearance. This variability is a recognized practical feature of multistep/one-step trichrome procedures and does not affect fiber identification or interpretation of the collagen signal [23].

The sampling strategy entailed collecting multiple micrographs of the same region of interest using each staining method, as described in detail in Kolinko et al. [24]. We used one section per specimen and staining method. Using the ×20 objective, we captured 10–14 micrographs per section to cover the entire section area. Using a standardized and established stereological method based on Cavalieri’s principle, we quantified the volume fractions of individual components (skeletal muscle, smooth muscle, elastin, collagen, and adipose cells) in three regions (R1, R2, and R3) of the ewe perineal body. This method, based on the number of intersections between the test point grid and the object of interest [21, 25], is integrated into the Ellipse 3D software package (ViDiTo, Inc., Košice, Slovak Republic). An unbiased point grid of crosses was superimposed on an image of each sampled section. The points that land on the structure of interest (e.g., collagen) and the total number of points covering the entire region (the whole image, in our case) were counted. The area fraction was then calculated as the ratio of the number of points that hit the structure (e.g., collagen) to the total number of points that hit the whole region. This area fraction directly represents the volume fraction in 3D, assuming uniform sampling across the tissue. Other structures, such as the lumina of blood vessels, peripheral nerves, fibroblasts, and fibrocytes, were considered residual tissue. We calculated this residual tissue as 1 minus the sum of the quantified volume fractions.

The total number of intersections between the objects and the test point grid was 35,319. To avoid errors due to staining artifacts and uneven staining of skeletal muscle fibers in some specimens, a single observer (PK) performed the histological quantification manually.

### Statistical Analysis

We tested the normality of the volume fractions and mechanical parameters using the Shapiro–Wilk W test. All the statistical tests were nonparametric because some data sets did not pass the normality test. The statistical analysis was performed using Statistica software (version 8.0, Statsoft, Inc., Tulsa, OK, USA). Differences in structural and mechanical parameters among regions (R1, R2, and R3) were analyzed using a Friedman analysis of variance (ANOVA). Post hoc multiple comparisons were then performed using the Wilcoxon matched-pairs test to compare region pairs (R1 versus R2, R1 versus R3, and R2 versus R3). Differences in structural and mechanical parameters between groups (the POST-ERT group and the POST group) were analyzed using the Mann–Whitney *U* test. Average volume fractions for each component and mechanical parameters were calculated for all three perineal regions of the POST-ERT and POST groups and then used in the Mann–Whitney *U* test. The effect size was evaluated using the Statistica software and the Effect Size Calculator for Nonparametric Tests [26]: Kendall’s coefficient of concordance W (Friedman ANOVA test); η2 and d_Cohen_ values (Wilcoxon matched-pairs and Mann–Whitney *U* test).

## Results

In the present study, systemic estrogen exposure was ensured by the implantation of a sustained-release estradiol implant at the start of the intervention period. Although circulating estradiol (E2) serum levels were not measured directly, effective estrogenization was confirmed by multiple well-established biological and functional surrogate markers. Specifically, uterine size was assessed at necropsy. Estrogen-treated ewes in the POST-ERT group demonstrated a significantly greater increase in vaginal epithelial thickness (+202%) compared with estrogen-deficient POST group animals. They also showed restoration of the glycogen-positive epithelial layer, improved vaginal lubrication, reduced vaginal pH, increased uterine weight, and significantly higher vaginal compliance in biomechanical tests. These data have been reported elsewhere [20] and are consistent with robust systemic estrogen effects. They have also been previously validated in the ovine menopause model as reliable indicators of estrogen replacement efficacy [27].

The morphometric data are in the form of continuous variables provided in the supplementary material (Supplement 1; Tables 1, 2). Complete statistical analyses of the Friedman ANOVA test, Wilcoxon matched-pairs, and Mann–Whitney *U* tests are provided in Tables 3 and 4.

**Table 1:** Mechanical parameters: Young’s modulus of elasticity in the small deformation region (E_0_, MPa), Young’s modulus of elasticity in the large deformation region (E_1_, MPa), ultimate stress (MPa) at rupture, ultimate strain (%) at rupture of the perineal body of the postmenopausal ewes with delayed estrogen treatment (POST-ERT group) and postmenopausal ewes without estrogen treatment (POST group)

Parameter	POST-ERT group				POST group			
	R1(*n* = 8)	R2(*n* = 8)	R3(*n* = 8)	Average (*n* = 24)	R1(*n* = 7)	R2(*n* = 7)	R3(*n* = 7)	Average (*n* = 21)
E_0_ (MPa)	0.03 (0.02–0.05)	0.03 (0.01–0.05)	0.02 (0.02–0.05)	0.03 (0.02–0.05)	0.04 (0.02–0.11)	0.03 (0.02–0.04)	0.01 (0.01–0.03)	0.03 (0.01–0.04)
E_1_ (MPa)	0.32 (0.21–0.56)	0.41 (0.34–0.69)	0.38 (0.29–0.71)	0.38 (0.28–0.58)	0.25 (0.15–0.83)	0.63 (0.33–1.00)	0.27 (0.25–0.47)	0.33 (0.25–0.63)
Ultimate strain (%)	93 (59–105)	106 (86–152)	100 (79–115)	94 (80–119)	74 (58–114)	89 (72–114)	106 (73–123)	89 (72–114)
Ultimate stress (MPa)	0.17 (0.13–0.23)	0.30 (0.23–0.43)	0.22 (0.14–0.54)	0.23 (0.15–0.37)	0.15 (0.07–0.42)	0.31 (0.26–0.36)	0.21 (0.08–0.22)	0.23 (0.15–0.36)

Both groups were 280 days post-surgical ovariectomy

Regions R1–R3: R2 = the midline region, R1 = the sagittal section 5 mm to the left from the midline, R3 = the sagittal section 5 mm to the right from the midline

Given as the median (interquartile range)

The number of investigated specimens was 24 (8 per region) in the POST-ERT group and 21 (7 per region) in the POST group

**Table 2:** The volume fractions of the smooth-muscle tissue, skeletal-muscle tissue, collagen, elastin, adipose tissue, and residual tissue (neural cells, blood vessels, lumina, fibrocytes, and fibroblasts) along the perineal body of the postmenopausal ewes with estrogen treatment (POST-ERT group) and postmenopausal ewes without estrogen treatment (POST group)

Component	POST-ERT group				POST group			
	R1(*n* = 8)	R2(*n* = 7)	R3(*n* = 7)	Average (*n* = 22)	R1(*n* = 7)	R2(*n* = 7)	R3(*n* = 7)	Average (*n* = 21)
Smooth muscle	0.07 (0.0–0.18)	0.23 (0.17–0.27)	0.07 (0.01–0.16)	0.16 (0.05–0.23)	0.07 (0.03–0.12)	0.21 (0.04–0.30)	0.09 (0.04–0.35)	0.09 (0.04–0.22)
Skeletal muscle	0.10 (0.07–0.28)	0.04 (0.00–0.17)	0.24 (0.14–0.28)	0.15 (0.04–0.27)	0.14 (0.00–0.19)	0.21 (0.00–0.36)	0.20 (0.06–0.26)	0.17 (0.00–0.26)
Collagen	0.33 (0.29–0.38)	0.36 (0.30–0.37)	0.32 (0.26–0.40)	0.33 (0.30–0.37)	0.24 (0.15–0.34)	0.24 (0.18–0.33)	0.21 (0.14–0.27)	0.23 (0.17–0.27)
Elastin	0.07 (0.06–0.07)	0.07 (0.05–0.08)	0.06 (0.05–0.07)	0.06 (0.05–0.07)	0.05 (0.03–0.07)	0.05 (0.04–0.06)	0.04 (0.03–0.06)	0.05 (0.04–0.06)
Adipose cells	0.11 (0.06–0.16)	0.04 (0.01–0.06)	0.08 (0.04–0.12)	0.07 (0.04–0.12)	0.18 (0.06–0.64)	0.03 (0.02–0.05)	0.12 (0.07–0.22)	0.11 (0.03–0.19)
Residual tissue	0.22 (0.18–0.28)	0.22 (0.13–0.24)	0.24 (0.14–0.28)	0.22 (0.18–0.28)	0.20 (0.06–0.23)	0.25 (0.19–0.25)	0.3 (0.20–0.32)	0.24 (0.19–0.28)

Both groups were 280 days after surgical ovariectomy

Regions R1–R3: R2 = midline region, R1 = the sagittal section 5 mm to the left from the midline, R3 = the sagittal section 5 mm to the right from the midline

Given as the median (interquartile range)

The number of investigated specimens was 22 (7–8 per region) in the POST-ERT group and 21 (7 per region) in the POST group

**Table 3:** Statistical analysis of the mechanical and structural parameters of the perineal body of postmenopausal ewes

Variables	Friedman ANOVA, *p* (W)	Wilcoxon matched pairs test, *p* (η2; d_Cohen_)		
	R1, R2, R3	R1 vs R2	R1 vs R3	R2 vs R3
POST-ERT group				
E0	0.69 (0.05)	0.78 (< 0.01; 0.20)	0.40 (0.09; 0.62)	0.89 (< 0.01; 0.10)
E1	0.09 (0.30)	0.26 (0.16; 0.86)	0.09 (0.35; 1.48)	0.89 (< 0.01; 0.10)
Ultimate strain	0.32 (0.14)	0.21 (0.20; 1.00)	0.89 (< 0.01; 0.10)	0.33 (0.12; 0.74)
Ultimate stress	*0.04 (0.39)*	*0.01* (0.79; 3.93)	0.33 (0.12; 0.74)	1.0 (0; 0)
V_V_(smooth muscle)	*0.04 (0.53)*	0.13 (0.33; 1.40)	0.40 (0.10; 0.67)	*0.03* (0.81; 4.09)
V_V_(skeletal muscle)	0.11 (0.36)	0.06 (0.36; 1.50)	0.50 (0.07; 0.53)	0.07 (0.53; 2.12)
V_V_(collagen)	0.85 (0.03)	0.61 (0.04; 0.39)	0.74 (0.02; 0.26)	0.60 (0.05; 0.44)
V_V_(elastin)	0.31 (0.19)	0.87 (< 0.01; 0.13)	0.13 (0.33; 1.41)	0.60 (0.05; 0.44)
V_V_(adipose cells)	*0.04 (0.53)*	0.24 (0.20; 1.00)	0.40 (0.10; 0.67)	*0.03* (0.81; 4.09)
V_V_(residual tissue)	0.60 (0.08)	0.31 (0.15; 0.83)	0.50 (0.07; 0.53)	0.60 (0.05; 0.44)
POST group				
E0	0.16 (0.26)	0.18 (0.26; 1.19)	*0.03* (0.69; 2.98)	0.18 (0.26; 1.19)
E1	0.37 (0.14)	0.61 (0.04; 0.39)	0.61 (0.04; 0.39)	0.06 (0.49; 1.97)
Ultimate strain	0.56 (0.08)	0.18 (0.26; 1.19)	0.24 (0.20;1.0)	0.87 (< 0.01; 0.13)
Ultimate stress	0.28 (0.18)	0.50 (0.07; 0.53)	0.61 (0.04; 0.39)	*0.04* (0.59; 2.39)
V_V_(smooth muscle)	0.65 (0.06)	0.31 (0.15; 0.83)	0.74 (0.02; 0.26)	0.24 (0.20; 1.0)
V_V_(skeletal muscle)	0.34 (0.15)	0.35 (0.18; 0.93)	0.61 (0.04; 0.39)	0.87 (< 0.01; 0.13)
V_V_(collagen)	0.56 (0.08)	0.31 (0.15; 0.83)	1 (0; 0)	0.06 (0.50; 1.98)
V_V_(elastin)	0.77 (0.04)	1 (0; 0)	0.35 (0.18; 0.93)	0.61 (0.04; 0.39)
V_V_(adipose cells)	*0.03 (0.51)*	*0.02* (0.80; 4.00)	0.31 (0.15; 0.83)	0.06 (0.50; 1.98)
V_V_(residual tissue)	0.37 (0.14)	0.13 (0.33; 1.41)	0.06 (0.50; 1.98)	0.74 (0.02; 0.26)

POST-ERT group: postmenopausal ewes with delayed estrogen replacement therapy (ERT) in the midline region (R2) and in the sagittal section 5 mm to the left (R1) and 5 mm to the right (R3) from the midline (anatomical orientation)

POST group: postmenopausal ewes without ERT

E_0_: Young’s modulus in the small deformation region

E_1_: Young’s modulus in the large deformation region

V_V_(component): the volume fraction of a tissue component

Differences in structural and mechanical parameters between regions (R1, R2, and R3) were analyzed using the Friedman ANOVA test

The Wilcoxon matched-pairs test was used for post hoc multiple comparisons

The effect size was evaluated using Statistica software and the Effect Size Calculator for Nonparametric Tests: Kendall’s coefficient of concordance W (Friedman ANOVA test)

η2 and d_Cohen_ values (Wilcoxon matched-pairs)

*p* values < 0.05 are in italics

The interpretation is given by Cohen’s rule: r = 0.1 small effect, 0.3 = medium effect, >  = 0.5 large effect

**Table 4:** Statistical analysis of the mechanical and structural parameters of the perineal body of postmenopausal ewes

Variables	Mann Whitney *U* test, *p* (η2; d_Cohen_) POST-ERT vs POST			
	Average	R1	R2	R3
E0	0.63 (0.01; 0.15)	0.77 (0.01; 0.18)	0.95 (< 0.01; 0.06)	0.32 (0.07; 0.56)
E1	0.56 (0.01; 0.18)	0.86 (< 0.01; 0.12)	0.77 (< 0.01; 0.18)	0.22 (0.11; 0.70)
Ultimate strain	0.49 (0.01; 0.21)	0.86 (< 0.01; 0.12)	0.60 (0.02; 0.30)	0.95 (< 0.01; 0.06)
Ultimate stress	0.86 (< 0.01; 0.05)	0.95 (< 0.01; 0.06)	0.86 (< 0.01; 0.12)	0.45 (0.04; 0.43)
V_V_(smooth muscle)	0.96 (< 0.01; 0.02)	0.95 (< 0.01; 0.06)	0.61 (0.02; 0.31)	0.44 (0.05; 0.46)
V_V_(skeletal muscle)	0.97 (< 0.01; 0.02)	0.60 (0.02; 0.30)	0.44 (0.05; 0.46)	0.61 (0.02; 0.31)
V_V_(collagen)	* < 0.001* (0.32; 1.38)	0.06 (0.26; 1.18)	0.07 (0.25; 1.14)	*0.02* (0.44; 1.79)
V_V_(elastin)	* < 0.01* (0.17; 0.91)	0.16 (0.14; 0.81)	0.06 (0.28; 1.25)	0.31 (0.08; 0.61)
V_V_(adipose cells)	0.43 (0.02; 0.25)	0.22 (0.11; 0.70)	0.70 (0.01; 0.24)	0.25 (0.11; 0.69)
V_V_(residual tissue)	0.55 (0.01; 0.19)	0.27 (0.09; 0.63)	0.16 (0.15; 0.85)	0.20 (0.13; 0.77)

POST-ERT group: postmenopausal ewes with delayed estrogen replacement therapy (ERT) in the midline region (R2) and in the sagittal section 5 mm to the left (R1) and 5 mm to the right (R3) from the midline (anatomical orientation)

POST group: postmenopausal ewes without ERT

E_0_: Young’s modulus in the small deformation region

E_1_: Young’s modulus in the large deformation region

V_V_(component): the volume fraction of a tissue component

Differences in structural and mechanical parameters between groups (group POST-ERT and group POST) were analyzed using the Mann–Whitney *U* test

The effect size was evaluated using Statistica software and the Effect Size Calculator for Nonparametric Tests: Kendall’s coefficient of concordance W (Friedman ANOVA test); η2 and d_Cohen_ values (Mann–Whitney *U* test)

*p* values < 0.05 are in italics

The interpretation is given by Cohen’s rule: r = 0.1 small effect, 0.3 = medium effect, >  = 0.5 large effect

### Mechanical Measurements

**Fig. 2:**
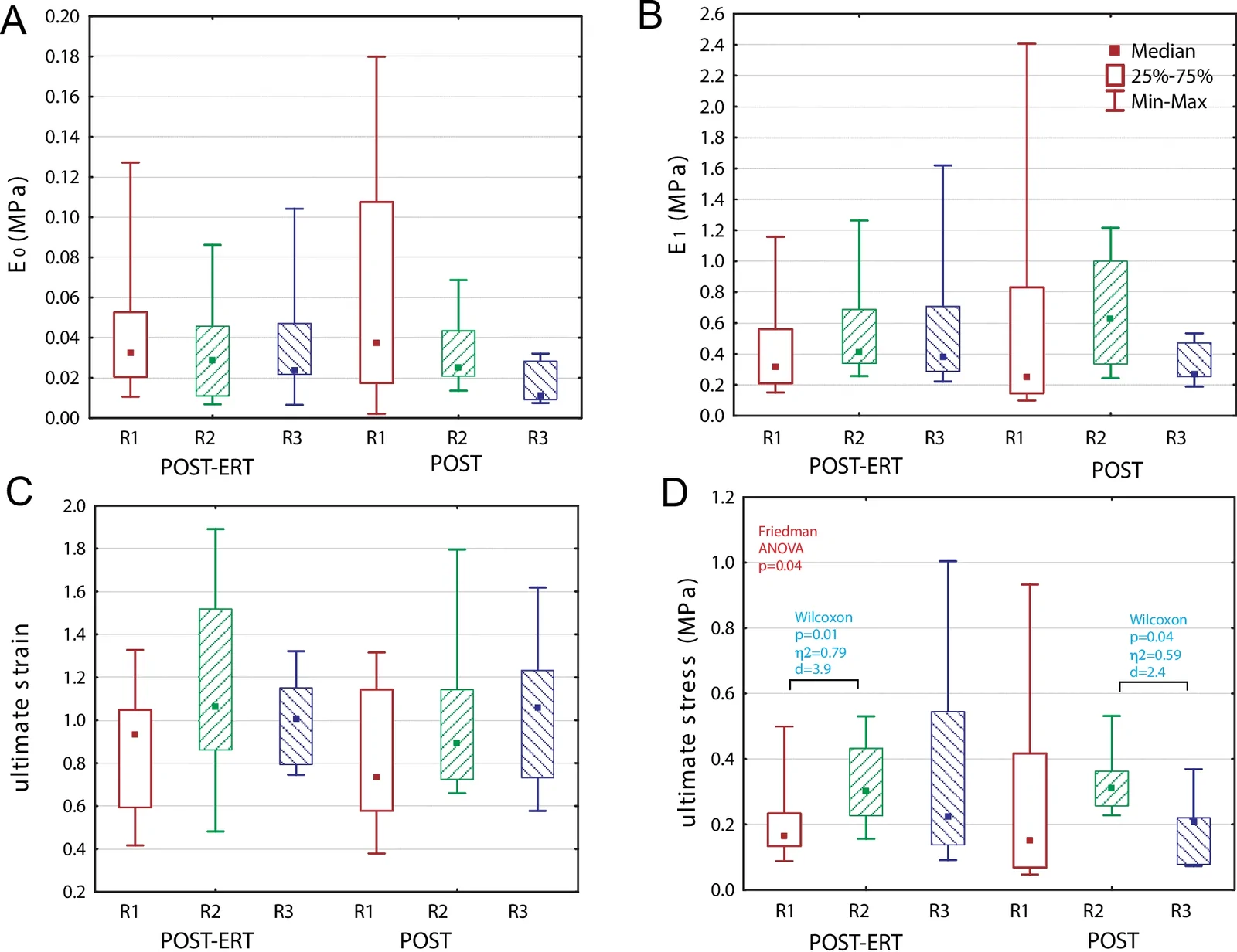
Mechanical parameters of the perineal body of postmenopausal ewes with (POST-ERT) and without (POST) delayed estrogen replacement therapy (ERT). **A** E_0_, Young’s modulus of elasticity in the small deformation region. **B** E_1_, Young’s modulus of elasticity in the large deformation region. **C** The ultimate stress when the rupture occurred. **D** The ultimate strain at rupture. Regions: R2—midline region, R1 and R3—sagittal regions. The *square dot* denotes the median, the *rectangle* spans the interquartile range, and the *whiskers* denote the limit values. Differences between regions were tested using the Friedman ANOVA test and the Wilcoxon matched-pairs test. Differences between groups were tested using the Mann–Whitney *U* test. The level of significance was 0.05

The mechanical parameters determined in the midline and sagittal regions, as well as the investigated groups, are shown in Table 1 and Fig. 2. For the POST-ERT group, the averaged mechanical parameters (and the interquartile range) from all three regions were Young’s modulus of elasticity at low deformation: 0.03 (0.02–0.05) MPa; Young’s modulus of elasticity at large deformation: 0.38 (0.28–0.58) MPa; ultimate stress: 0.23 (0.15–0.37) MPa; ultimate strain: 0.94 (0.80–1.19). For the POST group, the averaged mechanical parameters (and the interquartile range) from all three regions were Young’s modulus of elasticity at low deformation: 0.03 (0.01–0.04) MPa; Young’s modulus of elasticity at large deformation: 0.33 (0.25–0.63) MPa; ultimate stress: 0.23 (0.15–0.36) MPa; ultimate strain: 0.89 (0.72–1.14).

#### Regional Variability

In the POST-ERT group, the ultimate stress was higher in the midline R2 region (0.3 MPa) than in the sagittal R1 region (0.17 MPa) (*p* = 0.01). In the POST group, the ultimate stress was higher in the midline R2 region (0.31 MPa) than in the sagittal R3 region (0.21 MPa) (*p* = 0.04). There were no significant differences for any of the other parameters in either group (*p* > 0.05). The *p* values are shown in detail in Tables 3.

#### Group Differences

There was no significant difference in the mechanical parameters between the POST and POST-ERT groups based on the average values.

### Microscopic Quantification

**Fig. 3:**
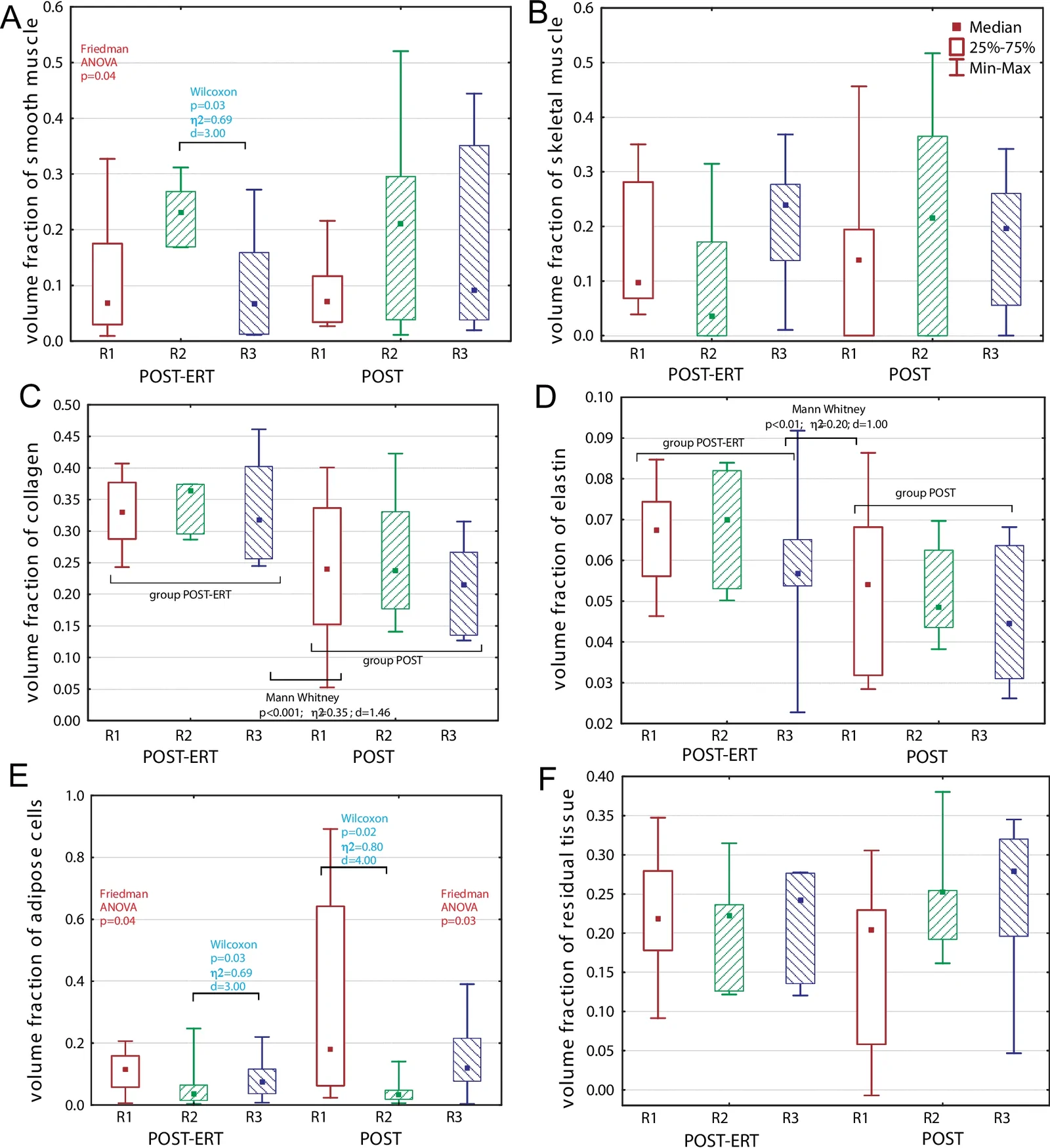
Volume fractions of smooth-muscle tissue (**A**), skeletal muscle tissue (**B**), collagen (**C**), elastin (**D**), adipose cells (**E**), and the residual tissue (**F**) along the perineal body of postmenopausal ewes with (POST-ERT) and without (POST) delayed estrogen replacement therapy (ERT). Regions: R2—midline region, R1 and R3—sagittal regions. The *square dot* denotes the median, the *rectangle* spans the interquartile range, and the *whiskers* denote the limit values. Differences between regions were tested using the Friedman ANOVA test and the Wilcoxon matched-pairs test. Differences between groups were tested using the Mann–Whitney *U* test. The level of significance was 0.05

Table 2 and Fig. 3 show the assessed volume fractions of individual components in various regions and groups. The volume fractions averaged over all three regions are shown below. The data are shown as median (interquartile range). The perineal body of the POST-ERT group was mostly collagen (33% [30–37%]), followed by residual tissue (22% [18–28%]), smooth muscle (16% [5–23%]), and skeletal muscle (15% [4–27%]). The smallest tissue amounts were occupied by adipose tissue (7% [4–12%]) and elastin (6% [5–7%]). A similar distribution of tissue components was observed in the POST group: collagen covered 23% (17–27%) of the total tissue volume, residual tissue covered 24% (19–28%), adipose cells covered 11% (3–19%), skeletal muscles covered 17% (0–26%), smooth muscles covered 9% (4–22%), and elastin covered 5% (4–6%).

#### Regional Variability

The volume fraction of adipose cells was lower in the midline R2 region (4%) than in the sagittal R3 region (8%; *p* = 0.03) in the POST-ERT group and lower in the midline R2 region (3%) than in the sagittal R1 region (18%; *p* = 0.02) in the POST group.

The smooth-muscle volume fraction was higher in the midline R2 region (23%) than in the sagittal R3 region (7%; *p* = 0.03) in the POST-ERT group. There were no significant differences for any of the other parameters in either group (*p* > 0.05). The *p* values are shown in detail in Tables 3.

#### Group Differences

Based on the average values, the POST group had significantly smaller amounts of collagen (*p* < 0.001; 23% in the POST group versus 33% in the POST-ERT group) and elastin (*p* < 0.01; 5% in the POST group versus 6% in the POST-ERT group) than the POST-ERT group. The other volume fractions did not differ significantly (*p* > 0.05). The *p* values are shown in detail in Tables 4.

## Discussion

### Principal Findings

In this study, we found that the perineal body of postmenopausal ewes maintains mechanical integrity for up to 280 days after ovariectomy. Young’s moduli, ultimate stress, and ultimate strain were similar between estrogen-deficient (POST) and estrogen-treated (POST-ERT) animals. The only differences observed were histological: ERT treatment resulted in a 44% higher collagen fraction and a 20% higher elastin fraction than in untreated animals. Minimal regional variability was observed along the perineal body, suggesting structural and mechanical homogeneity after menopause.

### Mechanical Parameters

The mechanical changes caused by ovariectomy are altered over time. This finding is supported by comparing the obtained parameters with those from the study focused on early postmenopausal ewes (150 days after ovariectomy) [10]. Based on the supplement to the study by Kochová et al. [10] we calculated the average values for all specimens. The data are presented as the median (interquartile range): the Young’s modulus in the small deformation region was 0.01 (0.0–0.05) MPa; the Young’s modulus in the large deformation region was 1.07 (0.80–2.07) MPa; the ultimate stress was 0.59 (0.36–0.72) MPa; and the ultimate strain was 0.87 (0.58–1.05). Examining the data from the study by Kochová et al. [10] revealed that stiffness in the large deformation region E_1_ was higher 150 days after ovariectomy than 280 days after ovariectomy. A similar influence of the postmenopausal period was observed for ultimate stress. Evidently, the mechanical stiffness and ultimate stress of the perineal tissue decrease during the postmenopausal period, indicating that the tissue becomes more compliant.

Delayed ERT with an onset 180 days after ovariectomy and a duration of 100 days did not improve the mechanical properties, although it led to an increase in elastin and collagen volume fraction. We hypothesize that to have a more pronounced effect, the therapy must begin without undue delay as soon as possible after the onset of menopause or it should have a longer duration. However, these assumptions must be confirmed by further studies focusing on the differences in structure and mechanical properties influenced by the onset and duration of ERT. Moreover, although we quantified the volume fractions of tissue components, we did not capture matrix architecture and quality, such as fiber orientation/anisotropy, collagen crimp, crosslinking, elastin integrity, or hydration. These parameters are also key determinants of uniaxial tensile behavior [28–30]. Therefore, the volume fractions differences observed after delayed ERT may be due to remodeling that is insufficient in magnitude, is counterbalanced by other microstructural factors, or is not detectable by the current uniaxial tensile testing configuration.

The results for the POST group ewes were very close to the mechanical parameters obtained for postmenopausal human perineal tissue [9]: E_0_ = 0.02 (0.01–0.03) MPa (median (interquartile range); E_1_ = 0.23 (0.15–0.31) MPa; ultimate stress = 0.17 (0.10–0.30) MPa; and ultimate strain = 1.40 (0.8–1.6). Comparative data showing that postmenopausal ewe perineal tissues exhibit very similar mechanical properties to those of human postmenopausal tissues have significant clinical implications. We can assume that ovariectomy without ERT simulates the estrogen-deficient state of human menopause. This validates the ewe as a robust model for studying pelvic floor mechanics, tissue degeneration, surgical testing, and regenerative strategies within the context of menopause.

### Structural Parameters

Based on the supplement to the study [10], which provided the values of the structural parameters of the individual specimens 150 days after ovariectomy, we were able to calculate the average volume fractions of the individual components. The volume fractions are shown as the median (interquartile range): collagen 23 (18–26)%, skeletal muscle 2 (0–6)%, elastin 7 (5–9)%, smooth muscle 14 (7–17)%, adipose cells 6 (4–10)%, and residual tissue 49 (45–53)%.

The amount of collagen remained consistent in the postmenopausal group 150 and 280 days after ovariectomy. ERT led to an increase in collagen content.

The skeletal muscle content was significantly lower in the postmenopausal group 150 days after ovariectomy than 280 days afterward. ERT led to a slight decrease in skeletal muscle volume.

Elastin volume decreased 280 days after ovariectomy compared with the postmenopausal group 150 days after ovariectomy. ERT led to an increase in total tissue volume. The changes in elastin volume between 150 and 280 days after ovariectomy could be due to elastin degradation caused by chronic hypoestrogenism [31].

The structural parameters of the postmenopausal group, 280 days after ovariectomy, are, with the exception of skeletal muscle (lower for human tissue) and adipose cells (higher for human tissue), close to the parameters obtained for postmenopausal human perineal tissue [9]. The human volume fractions, median (interquartile range) were as follows: smooth muscle was 11 (3–17)%, skeletal muscle 3 (1–8)%, collagen 29 (15–39)%, elastin 7 (6–10)%, and adipose cells 27 (7–42)%. The close resemblance of most structural parameters between postmenopausal human and ewe perineal tissue, except for skeletal muscle and adipose content, supports the clinical validity of ewe models in pelvic floor research. This comparative analysis is crucial for translational studies of POP, perineal repair strategies, and biomaterials testing, in which mimicking postmenopausal tissue environments is essential. The reduced volume of perineal skeletal muscle in humans versus ewes indicates a higher vulnerability to pelvic floor dysfunction, particularly in sedentary or aging populations [32]. Adipose infiltration, which was more prevalent in human tissue, contributes to tissue fragility, impaired healing, and increased laxity [15].

High stress is predominantly localized in the area close to the perineal body, indicating its significance as the primary site of obstetric anal sphincter injury occurrence [33]. Therefore, it is important to include this tissue in pelvic floor modeling. Our results could serve as input parameters for finite element models of pelvic tissue prolapse, including the perineal body [4, 34–36].

### Strengths and Limitations

A major strength of this study is its combination of detailed stereological analysis and standardized biomechanical testing across multiple perineal regions. Limitations include the small sample size and the lack of correction for multiple statistical comparisons, which reduces the ability to detect small, yet potentially clinically significant, effects. Additionally, the ERT regimen was relatively short (100 days) and began late, which may have reduced the potential effects of the treatment. Future studies should investigate the effects of an earlier initiation and longer duration of ERT.

### Potential Clinical Value

Our findings identify clinically relevant changes in the perineal body following menopause. In reproductive-aged women, the perineal body is a heterogeneous structure that anchors several pelvic floor muscles. After menopause, this heterogeneity is reduced, and the tissue appears more uniform. Such structural changes may be associated with pelvic floor dysfunction and increased susceptibility to mechanical injury [37, 38]. Conversely, greater uniformity may result in more consistent tensile properties and load distribution, which could be relevant for surgical planning and perineal repair [39]. Estrogen replacement therapy initiated 180 days after ovariectomy and continued for 100 days produced only modest increases in collagen and elastin content, but only nonsignificant changes in mechanical properties. This suggests that the timing or duration of hormone therapy might influence tissue outcomes.

Comparisons with short-term postmenopausal ewes indicate that menopausal changes in tissue composition and biomechanics progress over time. The observed similarities between postmenopausal ewe and human perineal tissue support the use of the ewe as a translational model for investigating pelvic floor mechanics and evaluating surgical or regenerative interventions.

The findings from the ewe model are consistent with current clinical evidence on estrogen replacement in postmenopausal women. Estrogen is known to improve vaginal tissue quality by enhancing epithelial integrity and vascularity [11, 40]; however, there is no strong evidence that it alone can prevent or reverse POP [41]. Similarly, although vaginal estrogen may improve urinary urgency and frequency, its effect on stress urinary incontinence is inconsistent [40].

Emerging evidence suggests that earlier initiation of estrogen therapy might offer greater benefit, although this remains limited [42, 43]. In this context, the present findings reinforce that although hormonal factors contribute to tissue quality, they are insufficient to restore structural support. As suggested in the introduction, this highlights a potentially greater role for the perineal body in maintaining pelvic floor integrity.

## Conclusion

This study shows that, even after 280 days of estrogen deprivation, the perineal body of the ewe retains uniform mechanical strength. Delayed ERT modestly increases collagen and elastin content but does not significantly alter mechanical stiffness or rupture resistance. These results suggest that late-onset ERT might have a limited ability to restore perineal mechanical function. This emphasizes the importance of early or prolonged intervention in clinical settings.

The close similarity between postmenopausal ewe and human perineal tissue validates the use of ewes as an experimental model for pelvic floor mechanics. The quantitative mechanical and histological data reported here could serve as input parameters for finite element models used to study POP and perineal repair in postmenopausal women.

## Supplementary Information

Below is the link to the electronic supplementary material.

Supplement 1 (Table): Morphometric data in the form of continuous variables of the perineal body of postmenopausal ewes with (POST-ERT) and without (POST) delayed estrogen replacement therapy (ERT) in the midline region (R2) and in sagittal sections 5 mm to the left (R1) and 5 mm to the right (R3) from the midline. The data include the volume fractions of smooth-muscle tissue, skeletal-muscle tissue, collagen, elastin, adipose cells, residual tissue (blood vessels, peripheral nerve cells, fibroblasts, and fibrocytes), intervals of small (strain region 1) and large (strain region 2) deformation regions, Young’s modulus in small (E0) and large (E1) deformation regions, ultimate stress, and ultimate strain. Twenty-four specimens underwent mechanical testing and 22 underwent structure quantification in the POST-ERT group, and 21 specimens underwent mechanical testing and 21 underwent structure quantification in the POST group (XLS 22 KB)

## Data Availability

Data are available upon reasonable request.
